# MiR-216a-5p protects against high glucose-induced HMC injury by targeting the HMGB1/RAGE signaling pathway

**DOI:** 10.3389/fendo.2025.1669791

**Published:** 2025-10-14

**Authors:** Juan Zhang, Xiyin Zheng, Hong Zhang, Juan Chen

**Affiliations:** Department of Endocrinology, The Affiliated Huai’an No.1 People’s Hospital of Nanjing Medical University, Huai’an, Jiangsu, China

**Keywords:** miR-216a-5p, diabetic nephropathy, high-mobility group box-1, receptor for advanced glycation end-products, signaling pathway

## Abstract

The pathogenesis of diabetic nephropathy (DN), a primary microvascular complication of diabetes and a leading cause of end-stage renal disease, remains incompletely understood. This study delved into the role and underlying mechanisms of miR-216a-5p in the development of DN. Our initial findings revealed a lower serum level of miR-216a-5p in DN patients (*P* < 0.05). *In vitro* experiments, in which high glucose concentrations were used to stimulate human mesangial cells (HMCs), demonstrated a significant increase in the protein level of high mobility group box 1 (HMGB1) and a marked decrease in miR-216a-5p expression (all *P* < 0.05). Subsequent cell experiments showed that miR-216a-5p enhanced HMC viability, stimulated cell proliferation and inhibited cell apoptosis. It also alleviated the fibrosis and inflammatory response of HMC cells under high glucose conditions (all *P* < 0.05). A dual-luciferase reporter assay confirmed a direct binding between HMGB1 and miR-216a-5p. Moreover, miR-216a-5p suppressed the expression of HMGB1, as well as its receptor for advanced glycation end products (RAGEs). In summary, miR-216a-5p protects against high glucose-induced HMC injury by targeting the HMGB1/RAGE pathway, providing a new perspective for the subsequent treatment of DN.

## 1 Introduction

Diabetic nephropathy (DN) is a common microvascular complication of diabetes. As the population of diabetes expands, the prevalence of DN has steadily rose to 25–35%, making DN a primary etiological factor for end-stage renal disease (ESRD) (1, 2). The pathology of DN is hallmarked by the proliferation of human mesangial cells (HMCs), excessive deposition of extracellular matrix (ECM), and progressive fibrosis, all of which ultimately culminate in glomerulosclerosis (3, 4). In addition to hemodynamic and metabolic disturbances, hyperglycemia also induces other biochemical changes, such as protein glycation, methylglyoxal accumulation and AGE formation, which aggravate microvascular damage and immune dysregulation in diabetes, further driving the progression of DN (5). Nevertheless, the molecular regulatory networks underlying HMC injury should be further elucidated to identify novel therapeutic targets.

The receptor for advanced glycation end-products (RAGE) and its ligand high-mobility group box-1 (HMGB1) are linked into a central signaling axis in the progression of DN (6–8). In a high-glucose environment, the expression of HMGB1 is upregulated to activate the NF-κB and MAPK pathways downstream RAGE, which in turn promotes the secretion of pro-inflammatory cytokines, such as TNF-α and IL-6, and induces oxidative stress injury (9, 10). Our previous clinical studies have documented a significant increase in the serum HMGB1 level of DN patients, and this level correlates positively with the urinary protein excretion rate (11). However, the role and post-transcriptional regulatory mechanisms of HMGB1 in HMC damage remain unclear.

Recent studies have revealed that miRNAs, which modulate gene expression by targeting the 3’UTR of mRNAs, play a significant role in DN (12, 13). For instance, miR-214 targets DIAPH1 to alleviate renal fibrosis and inflammatory responses (13), and miR-203 reduces renal cell injury in DN by regulating SOCS (14). Under a high-glucose stimulation, miR-216a-5p can target FoxO1 to promote the proliferation of renal mesangial cells (15). Chen et al. also confirmed that Rg3 can attenuate diabetic kidney disease progression by downregulating miR-216a-5p (16). Although miR-216a-5p has been implicated in renal mesangial cell proliferation, its role in DN and the activation of HMGB1/RAGE signaling is still elusive.

Bioinformatic analyses have revealed a high complementarity between the seed sequence of miR-216a-5p and the 3’UTR of HMGB1 mRNA. We hypothesized that miR-216a-5p might target HMGB1 to block RAGE signaling, thus alleviating high glucose (HG)-induced inflammation, oxidative stress, and fibrosis in HMCs. Although this study has certain limitations, it first systematically confirms the regulatory role of the miR-216a-5p/HMGB1 axis in DN, providing a new clue for the clinical treatment of DN.

## 2 Materials and methods

### 2.1 Materials

The HMC cells were purchased from the American Type Culture Collection (ATCC, Manassas, VA, USA). miR-216a-5p mimic, miR-216a-5p inhibitor, mimic NC, inhibitor NC, siNC, siHMGB1 were synthesized by Ribobio (Guangzhou, China). For detailed information on the reagents used in the research, please refer to the supplementary information.

### 2.2 Serum sample collection

After fasting for 8 to 10 hours, venous blood was collected from the patient in the morning, centrifuged for 3000r at room temperature for 10 minutes. The supernatant was collected into an EP tube and stored in a -80°C refrigerator to establish the corresponding clinical specimen bank. Ultimately, according to the clinical medical record data, the included population was divided into the healthy control group (n=9), the diabetes mellitus (DM) group (n=16), and the DN group (n=21) in sequence. For specific information, please refer to the supplementary document.

### 2.3 ELISA

The serum levels of HMGB1, serum inflammatory factors, IL-1β, IL-6, TNF-α and MCP-1 in each group were detected in accordance with the instructions of kit manufacturers. One duplicate well was set for all samples during testing, and the test results were all within the range of the standard curve (R^2^ > 0.99).

### 2.4 Cell culture and transfection

HMCs were cultured in the DMEM supplemented with 10% FBS and 1% Penicillin/Streptomycin (P/S) at 37°C in an environment with 5% CO2. According to the manufacturer’s protocol, the miR-216a-5p mimics, inhibitors and corresponding controls were transfected into HMCs using Lipofectamine 3000 (Invitrogen Corporation, USA). After transfection for 6 hours, Lipofectamine 3000 was replaced with fresh culture medium. The cells were treated with 30 mmol/L glucose for 48 hours to simulate an HG environment, and the HMCs cultured with 5.5 mmol/L normal glucose (NG) were set as the control group. In addition, mannitol (final concentration 25.5mM) was added to the basic DMEM medium to make the osmotic pressure consistent with that of 30mM high glucose medium (excluding the effect of osmotic pressure on HMC cells), and then stored at 4°C. The cell experiments covered the NG group, the HG group, the HG+mimic NC group, the HG+miR-216a-5p mimic group, the HG+inhibitor NC group, and the HG+miR-216a-5p inhibitor group. siHMGB1 and the corresponding controls (siHMGB1 RNA sequence: 5’-CCCGUUAUGAAAGAGAAAUTT-3’, 5’-AUUUCUCUUUCAUAACGGGTT-3’; siNC RNA sequence: 5’-UUCUCCGAACGUGUCACGUTT-3’, 5’-ACGUGACACGUUCGGAGAATT-3’) were co-transfected into HMCs with miR-216a-5p mimics and corresponding controls.

### 2.5 CCK-8 assay

After the cells were grouped and treated, the viability of HMCs was detected by the CCK-8 method. In each group, the cells were inoculated into 96-well plates (1×10^4^ cells per well). Then, 100 μL of medium containing 10 μL of CCK-8 reagent was added. After incubation at 37°C for 2 hours, the OD value at 450 nm in each well was determined by a microplate reader.

### 2.6 Cell proliferation detection

Cells were treated using the EdU kit according to the instructions of the reagent supplier. Subsequently, the cells were fixed with 4% paraformaldehyde, treated with 0.5% Triton X-100 (to increase the permeability of the cell membrane), incubated with the prepared Apollo staining reaction solution, and then stained with the DAPI reaction solution. EdU was used to label DNA glow. Finally, the cell proliferation was observed under a fluorescence microscope and quantitatively analyzed using Image-J software.

### 2.7 Cell apoptosis detection

After treatment, the cells were gently washed with PBS, digested with trypsin, and centrifuged. The supernatant was discarded, and the cells were suspended in the combined buffer solution. The cell suspension was added with Annexin V-FITC and propidium iodide (PI), gently mixed, incubated in the dark at room temperature for 15 minutes. The apoptosis of cells was detected by flow cytometry.

### 2.8 Dual-luciferase reporter gene assay

To detect the activity of luciferase, dual luciferase reporter vectors of wild type (WT) and mutant type (MUT) of HMGB1 were constructed. Putative sites of 3’-UTR of HMGB1 and miR-216a-5p were described by sequence alignment in NCBI. WT or MUT 3’-UTR of HMGB1 were amplified and cloned into pmirGLO vectors (Promega, Madison, WI, USA) to obtain wt or mut luciferase report vectors, respectively. Then the reporter gene plasmid was co-transfected with miR-216a-5p mimic or miR-216a-5p NC into HMC cells using Lipofectamin 3000 according to the manufacturer’s protocols. The medium was changed at six hours after transfection. The cells were cultured in an incubator at 37°C for 48 hours and the luciferase activity was detected by an enzyme-labeled reader.

### 2.9 RNA extraction and reverse transcription quantification

Serum miRNA or total cellular RNA was extracted in accordance with the instructions of the kit. The RNA was detected for concentration and purity, and reversely transcribed into cDNA. Taking U6 as the internal reference gene, the expression level of miR-216a-5p was detected by qRT-PCR. Taking β-actin as the internal reference gene, the expression levels of IL-1β, IL-6, TNF-α and MCP-1 were detected by qRT-PCR. The 2- (ΔΔCT) method was used to calculate the relative expression level of each gene. The relevant PCR primers are listed in **Table 1**.

**Table 1:** Primer sequences.

Gene	Primer sequence (5’-3’)
miR-216a-5p	F:5’-ACACTCCAGCTGGGTATCTCAGCTGGC-3’ R:5’-CTCAACTGGTGTCGTGGA-3’
U6	F:5’-CTCGCTTCGGCAGCACA-3’ R:5’-AACGCTTCACGAATTTGCGT-3’
IL-1β	F:5’-GCCAACAAGTGGTATTCTCCA-3’ R:5’-TGCCGTCTTTCATCACACAG-3’
IL-6	F:5’-TTCTCCACAAGCGCCTTCGGTCCA-3’ R:5’-AGGGCTGAGATGCCGTCGAGGATGTA-3’
TNF-α	F:5’-GCTCCCTCTCATCAGTTCCA-3’ R:5’-GCTTGGTGGTTTGCTACGAC-3’
MCP-1	F:5’-CAGCCAGATGCAATCAATGCC-3’ R:5’-TGGAATCCTGAACCCACTTCT-3’
β-actin	F:5’-CATGTACGTTGCTATCCAGGC-3’ R:5’-CTCCTTAATGTCACGCACGAT-3’

### 2.10 Western blotting analysis

Fresh cell culture was homogenized in the ice-cold RIPA buffer (Beyotime Biotechnology, Shanghai, China). The supernatant was collected by centrifuging at 12–000 g at 4°C for 20 minutes, and the protein concentrations were quantitated by the BCA Protein Assay Kit (Thermo Fisher Scientific, Waltham, MA, USA). Every 20 ug of protein was subjected to polyacrylamide gel electrophoresis, membrane transfer, and sealed at room temperature with 5% skimmed milk for two hours. After incubation with the primary antibodies, including Col-IV (cAbam,1:2000), FN (Abcam,1:1000), TGF-β1 (Abcam,1:2000), and caspase-3 (Abcam,1: 1000), cleaved caspase-3 (Abcam,1:1000), HMGB1 (Abcam,1:2000), RAGE (Abcam,1:2000), GAPDH (Abcam,1:2000) overnight at 4°C, the membranes were washed, and then incubated with the corresponding HRP-labeled secondary antibody for two hours at room temperature. After washing, ECL was used for color development. The gray values of the protein bands were analyzed using the Image-J software.

### 2.11 Statistical analysis

Data were expressed as the mean ± standard deviation and analyzed using SPSS 27.0 or GraphPad Prism 9.0. One-way ANOVA analysis followed by Tukey’s multiple comparisons test was performed to analyze data of more than two groups. *P* < 0.05 was considered statistically significant.

## 3 Results

### 3.1 MiR-216a-5p expression is reduced in the serum of DN patients

**Figure 1:**
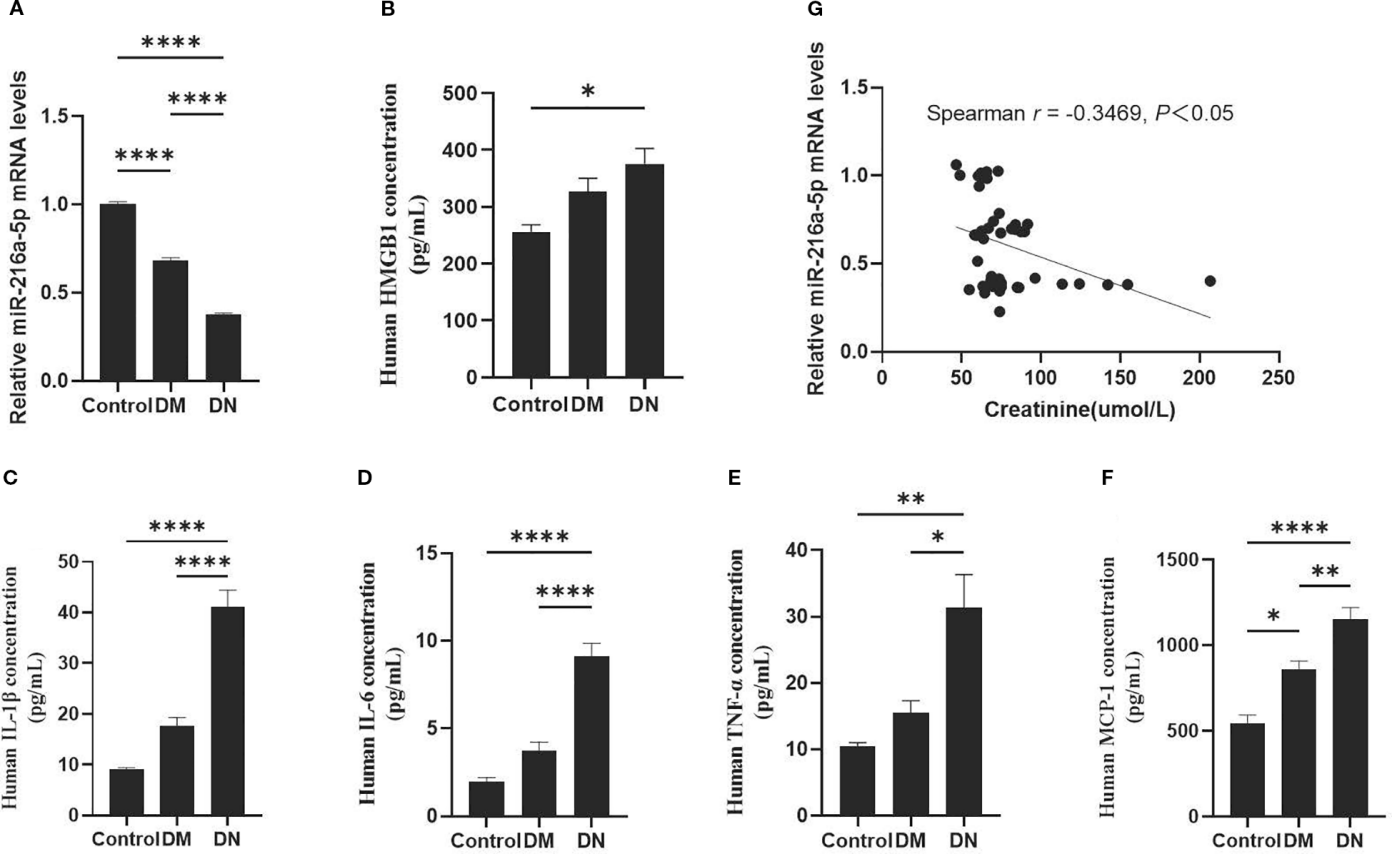
Expression of serum miR-216a-5p in DN patients and its correlation with HMGB1. According to the urinary albumin excretion rate, the included patients were divided into the healthy control group (n=9), the DM group (n=16), and the DN group (n=21). **(A)** RT-qPCR was used to analyze human serum miR-216a-5p. **(B)** Analysis of human serum HMGB1 by ELISA. **(C–F)** The serum levels of serum inflammatory factors IL-1β, IL-6, TNF-α and MCP-1 were detected by ELISA. **(G)** Correlation analysis of miR-216a-5p expression with creatinine in the serum. *P < 0.05, **P < 0.01, ****P < 0.0001 compared with control.

The level of serum miR-216a-5p decreased as the rate of urinary albumin excretion increased, while HMGB1 expression was upregulated in DN patients (**Figures 1A, B**). Serum levels of inflammatory factors IL-1β, IL-6, TNF-α, and MCP-1 were significantly elevated in DN patients, compared to those in normal patients (**Figures 1C–F**). Notably, the creatinine level of patients in the DN group was higher than that in the DM group, and the serum miR-216a-5p level was negatively correlated with the serum creatinine level (**Figure 1G**), indicating that miR-216a-5p may be involved in DN development.

### 3.2 MiR-216a-5p is downregulated and HMGB1 upregulated in HMCs stimulated with HG

**Figure 2:**
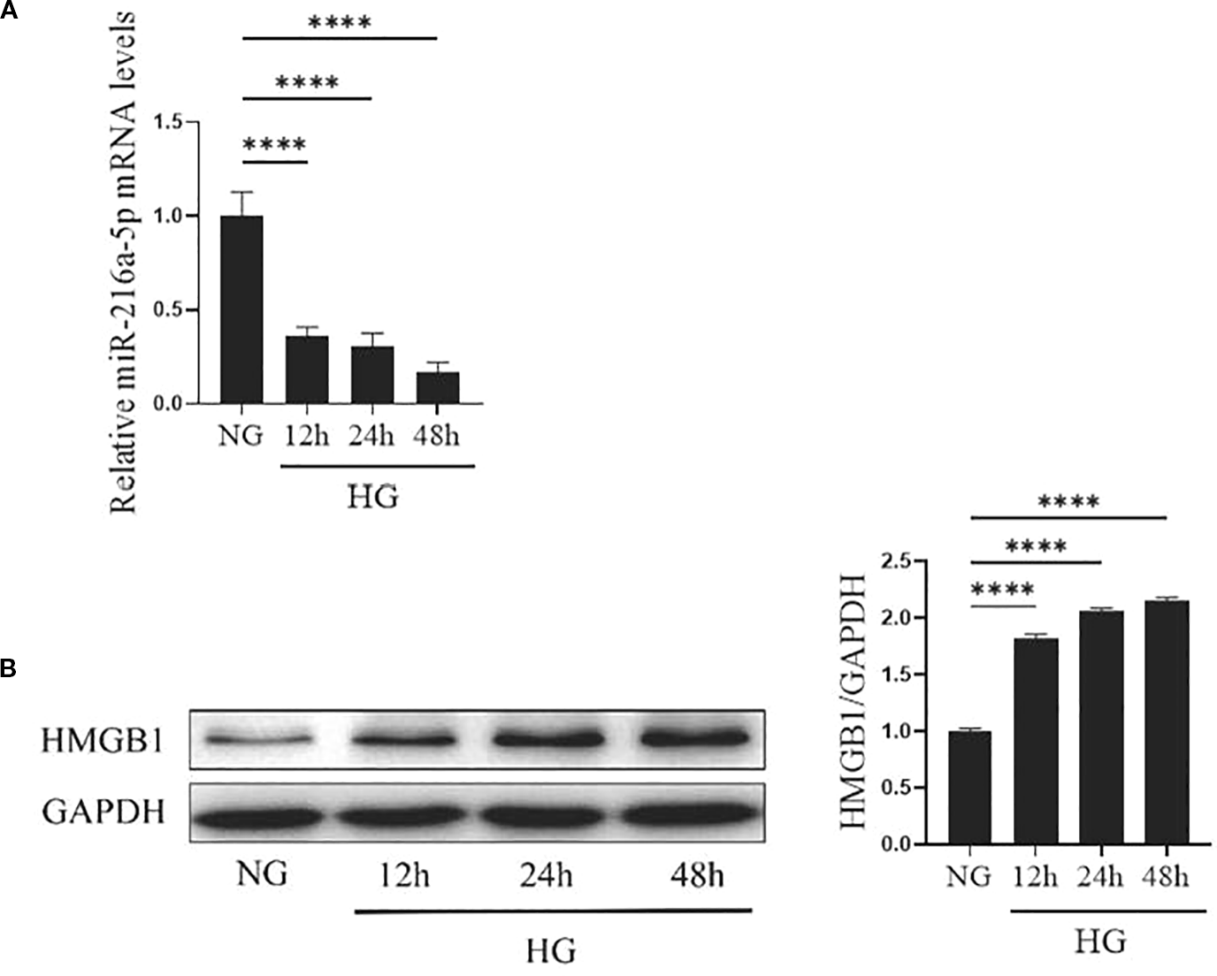
Expression of miR-216a-5p and HMGB1 in HMCs after HG stimulation. The levels of miR-216a-5p and HMGB1 in HMCs stimulated by HG at different time points were detected by RT-qPCR and Western blotting. **(A)** The expression of miR-216a-5p was analyzed by RT-qPCR. **(B)** Total protein was extracted from cells and the expression of HMGB1 was analyzed by Western blotting. Data were expressed as mean ± standard deviation, and analyzed using univariate ANOVA and Tukey multiple comparison test (n=3 per group). ****P < 0.0001, ^ns^ *P* > 0.05 compared with NG. NG, Normal glucose (5.5mmol/L) HG, high glucose (30 mmol/L).

At 12, 24, and 48 hours of high-glucose stimulation on HMCs, the expression of miR-216a-5p and HMGB1 was examined. As shown in **Figure 2**, the level of miR-216a-5p expression kept decreasing with the prolongation of high-glucose exposure, with the largest reduction observed at 48 hours (*P* < 0.05). The protein level of HMGB1 increased gradually with the duration of stimulation (*P* < 0.05). The opposing trends suggest that miR-216a-5p and HMGB1 play antagonistic roles in DN progression.

### 3.3 MiR-216a-5p enhances the viability, proliferation and attenuates the apoptosis of HMCs stimulated by HG

**Figure 3:**
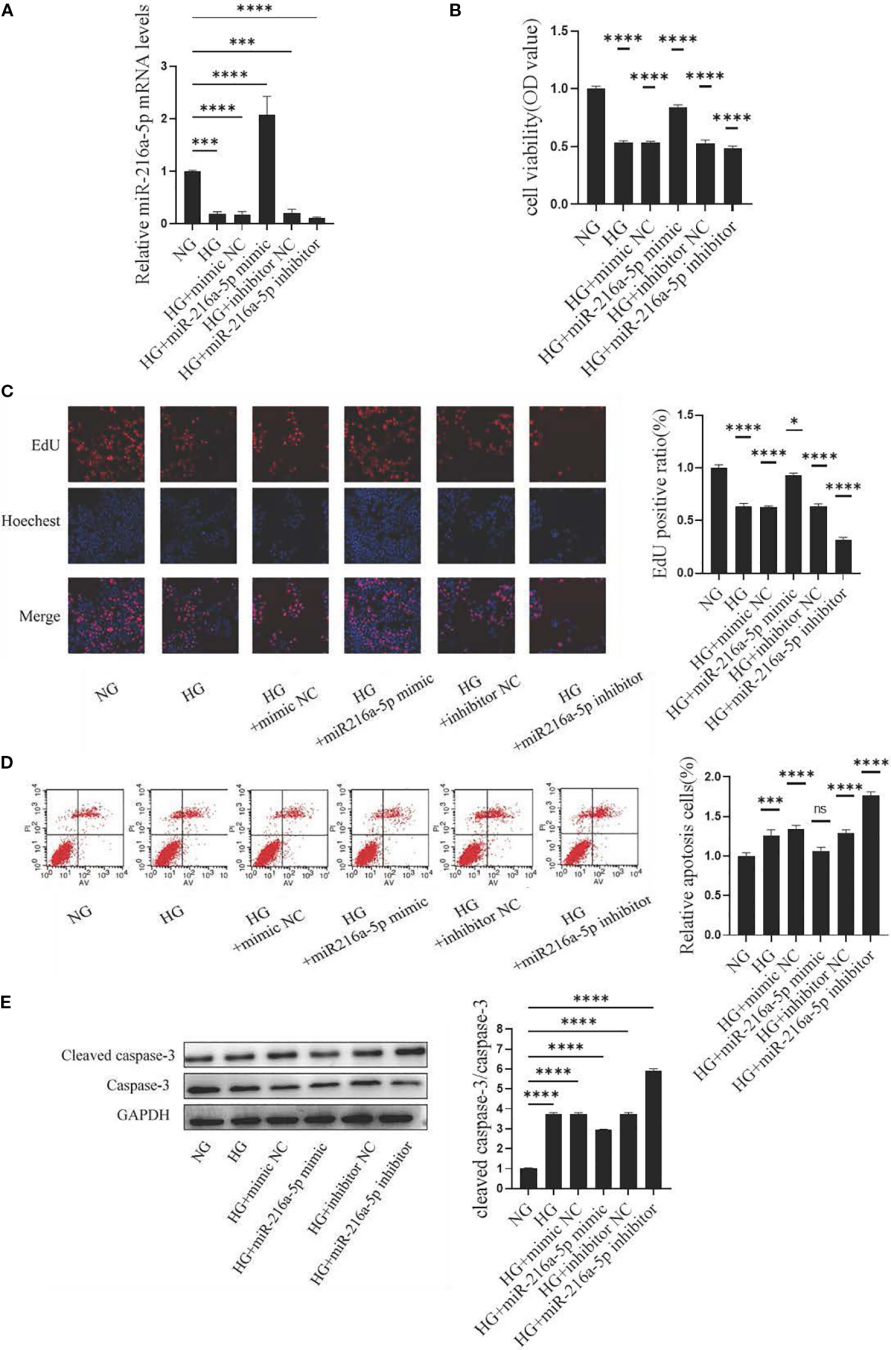
Effects of miR-216a-5p on the viability, proliferation and apoptosis of HMCs stimulated by HG. After overexpression and knockdown of miR-216a-5p, the viability, proliferation and apoptosis of HMCs were evaluated. **(A)** miR-216a-5p was analyzed by RT-qPCR (n=3 per group). **(B)** Cell viability was detected by the CCK8 method (n=7 per group). **(C)** Cell proliferation was observed by the EdU method (n=3 per group). **(D)** Flow cytometry analysis of apoptosis (n=3 per group). **(E)** Total protein was extracted from the cells and the expression of cleaved caspase-3 was analyzed by Western blotting (n=3 per group). The final transfection concentration of miRNA was 50 nM. Six hours post-transfection, the culture was continued for 24 hours when assessing the expression of miRNA-216a-5p, whereas it was maintained for 48 hours when evaluating the expression of the target gene or protein. Data were expressed as mean ± standard deviation. Statistical analysis was performed using univariate ANOVA and Tukey multiple comparison test. *P < 0.05, ***P < 0.001, ****P < 0.0001, ^ns^ *P* > 0.05 compared with NG. NG, Normal glucose (5.5 mmol/L) HG, high glucose (30 mmol/L).

We further clarified the role of miR-216a-5p in high glucose-induced HMC injury. PCR confirmed an efficient transfection of mimics or inhibitors (**Figure 3A**). CCK8 assays showed high-glucose stimulation reduced the viability of HMCs (*P* < 0.05), while mimics enhanced and inhibitors suppressed this viability (*P* < 0.05) (**Figure 3B**). EdU assays revealed a weaker proliferation in high-glucose groups (*P* < 0.05) (**Figure 3C**). Compared to controls, the mimic group had an enrichment (*P* < 0.05), and the inhibitor group had a paucity of EdU-positive cells (*P* < 0.05). Apoptosis assays indicated miR-216a-5p attenuated high glucose-induced apoptosis (*P* < 0.05) (**Figure 3D**). Western blotting showed miR-216a-5p decreased cleaved caspase-3 expression (*P* < 0.05) (**Figure 3E**). Overall, miR-216a-5p can exert a protective effect against high glucose-induced cytotoxicity.

### 3.4 MiR-216a-5p suppresses the inflammation and fibrosis of HMCs stimulated by HG

**Figure 4:**
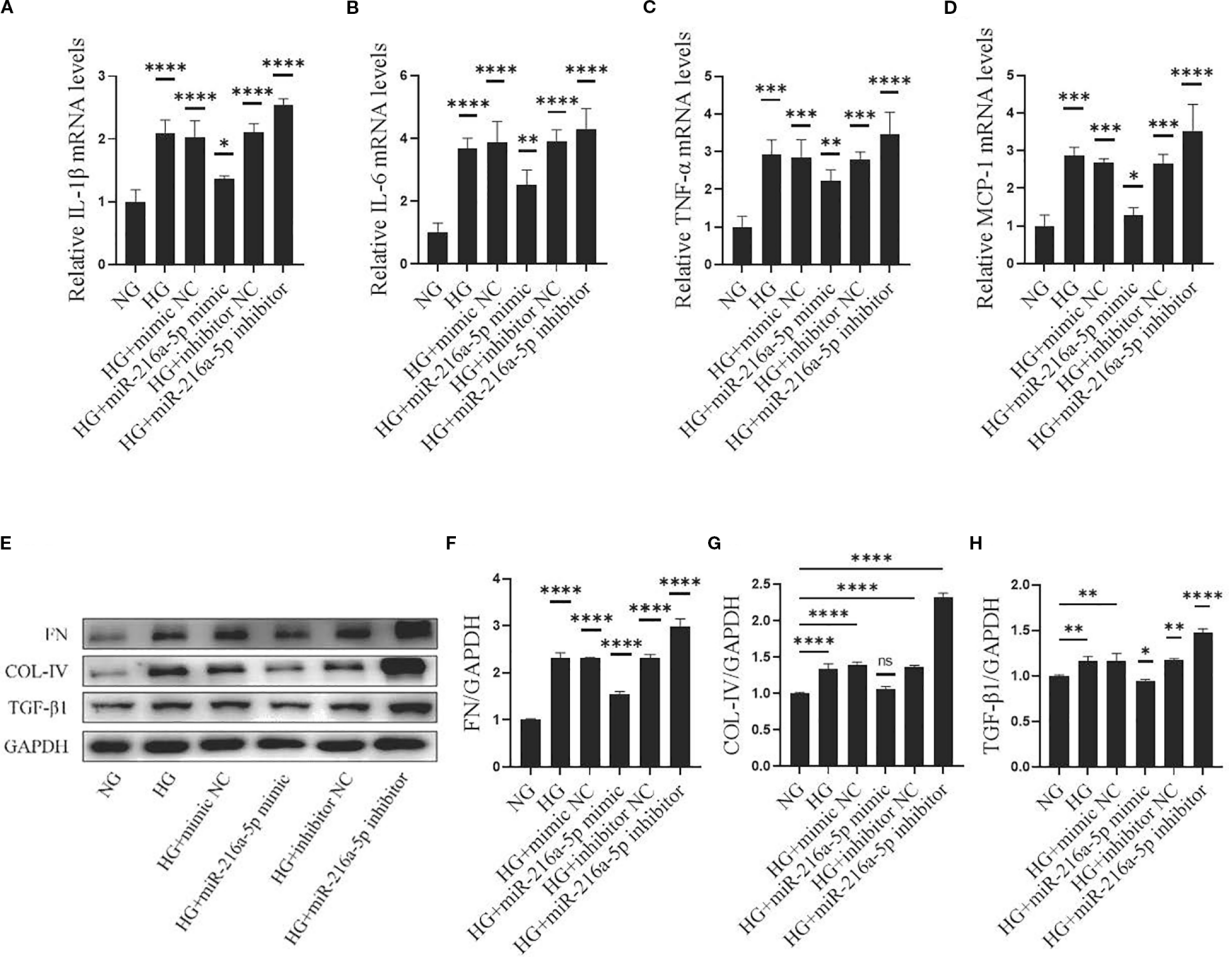
Effects of miR-216a-5p on inflammatory and fibrotic factors in HMCs stimulated by HG. After overexpression and knockdown of miR-216a-5p, the makers of inflammation and fibrosis were analyzed in HMCs stimulated by HG. **(A-D)** RT-qPCR was used to detect the expressions of inflammatory factors (IL-1β, IL-6, TNF-α and MCP-1). **(E-H)** Total cellular proteins were extracted, and fibrotic indicators (FN, COL-IV and TGF-β1) were analyzed by Western blotting. Data were expressed as mean ± standard deviation. Statistical analysis was performed using univariate ANOVA and Tukey multiple comparison test (n=3 per group). *P < 0.05, **P < 0.01, ***P < 0.001, ****P < 0.0001, ^ns^ *P* > 0.05 compared with NG. NG, Normal glucose (5.5 mmol/L) HG, high glucose (30 mmol/L).

Inflammatory responses are central to the development of DN. The mRNA levels of inflammation-related factors IL-1β, IL-6, TNF-α, and MCP-1 were analyzed (**Figures 4A–D**). In the HG group, the expression of these factors was significantly higher than that in the NG group (*P* < 0.05). Notably, these increases were attenuated by overexpressing miR-216a-5p (*P* < 0.05). Conversely, miR-216a-5p knockdown abolished the inhibition on inflammatory factors (*P* < 0.05). This suggests that miR-216a-5p can suppress the inflammation caused by sustained HG stimulation.

Fibrosis is a key link in DN progression. COL-IV, FN, and TGFβ1 are major components and regulators in the formation of ECM. In our study, HG significantly increased the protein levels of COL-IV, FN, and TGFβ1 in HMCs (**Figures 4E–H**) (all *P* < 0.05). Overexpressing miR-216a-5p reversed the increases of these markers, whereas knocking down miR-216a-5p led to their upregulation again (all *P* < 0.05), indicating that miR-216a-5p inhibits the fibrosis of HMCs.

### 3.5 MiR-216a-5p directly binds to the 3’UTR of HMGB1

**Figure 5:**
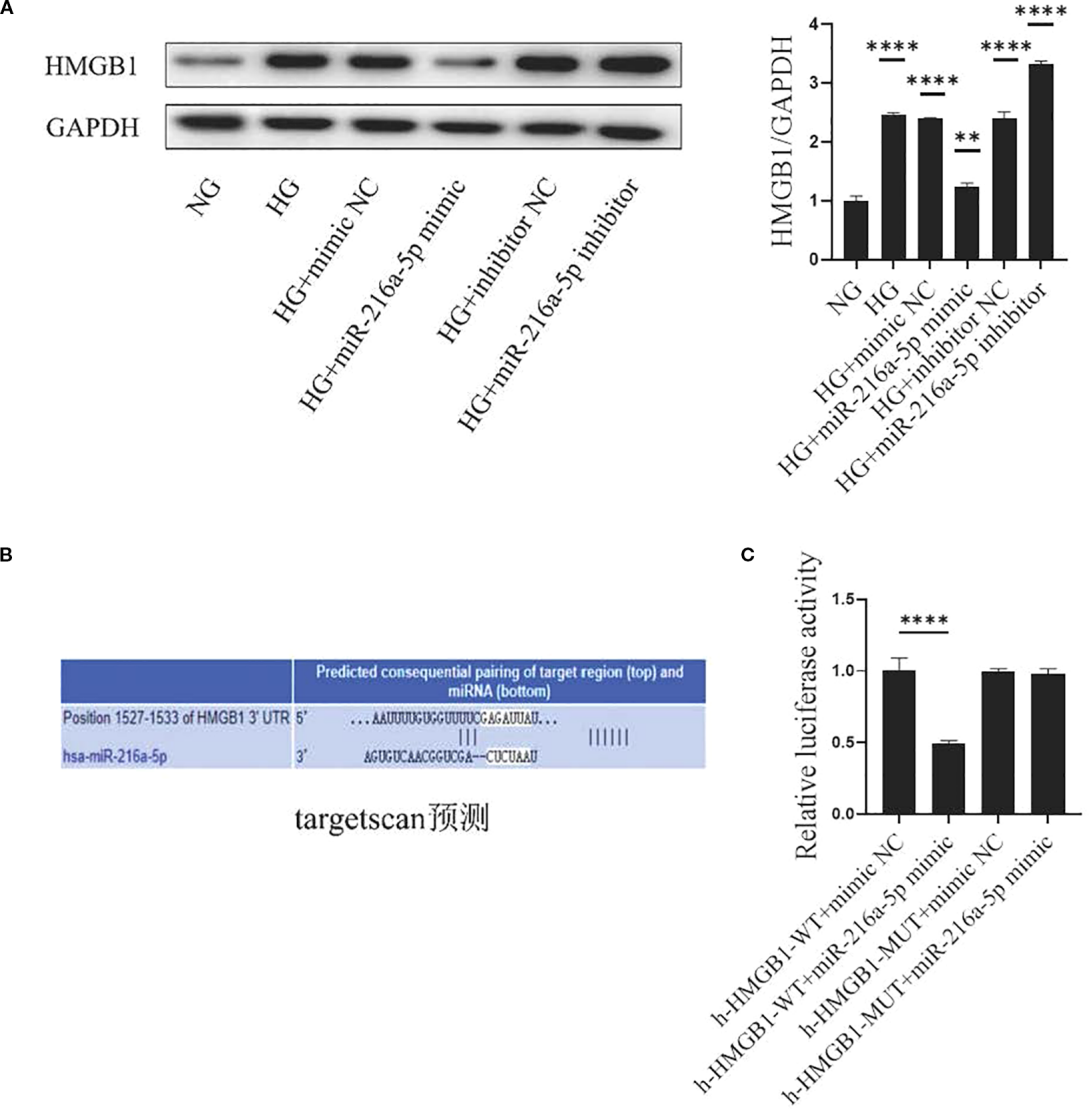
HMGB1 is the direct target of miR-216a-5p. **(A)** Extract the total protein of cells and analyze HMGB1 by Western blotting. **(B)** The bioinformatics software Target Scan predicts the miRNA of the HMGB1 target. **(C)** Dual-luciferase reporter gene detection of the miR-216a-5p action site. Data are expressed as mean ± standard deviation. Statistical analysis was performed using univariate ANOVA and Tukey multiple comparison test (n=3 per group). **P < 0.01, ****P < 0.0001, ^ns^ *P* > 0.05 compared with NG. NG, Normal glucose (5.5 mmol/L) HG, high glucose (30 mmol/L).

To explore how miR-216a-5p regulates HMGB1, we examined the effects of miR-216a-5p mimics or inhibitors on HMGB1 protein levels in HMCs treated with HG. miR-216a-5p overexpression reduced, while its knockdown increased the protein level of HMGB1 (**Figure 5A**). Bioinformatics analysis via TargetScan predicted miR-216a-5p as a potential HMGB1-targeting miRNA (**Figure 5B**). Dual-luciferase reporter assays (**Figure 5C**) showed that miR-216a-5p mimics significantly suppressed the luciferase activity of HMCs transfected with HMGB1-WT (*P* < 0.05), but not HMGB1-MUT (*P*>0.05). This implies that HMGB1 is likely a direct target of miR-216a-5p.

### 3.6 MiR-216a-5p targets HMGB1 to alleviate the injury of HMCs induced by HG

**Figure 6:**
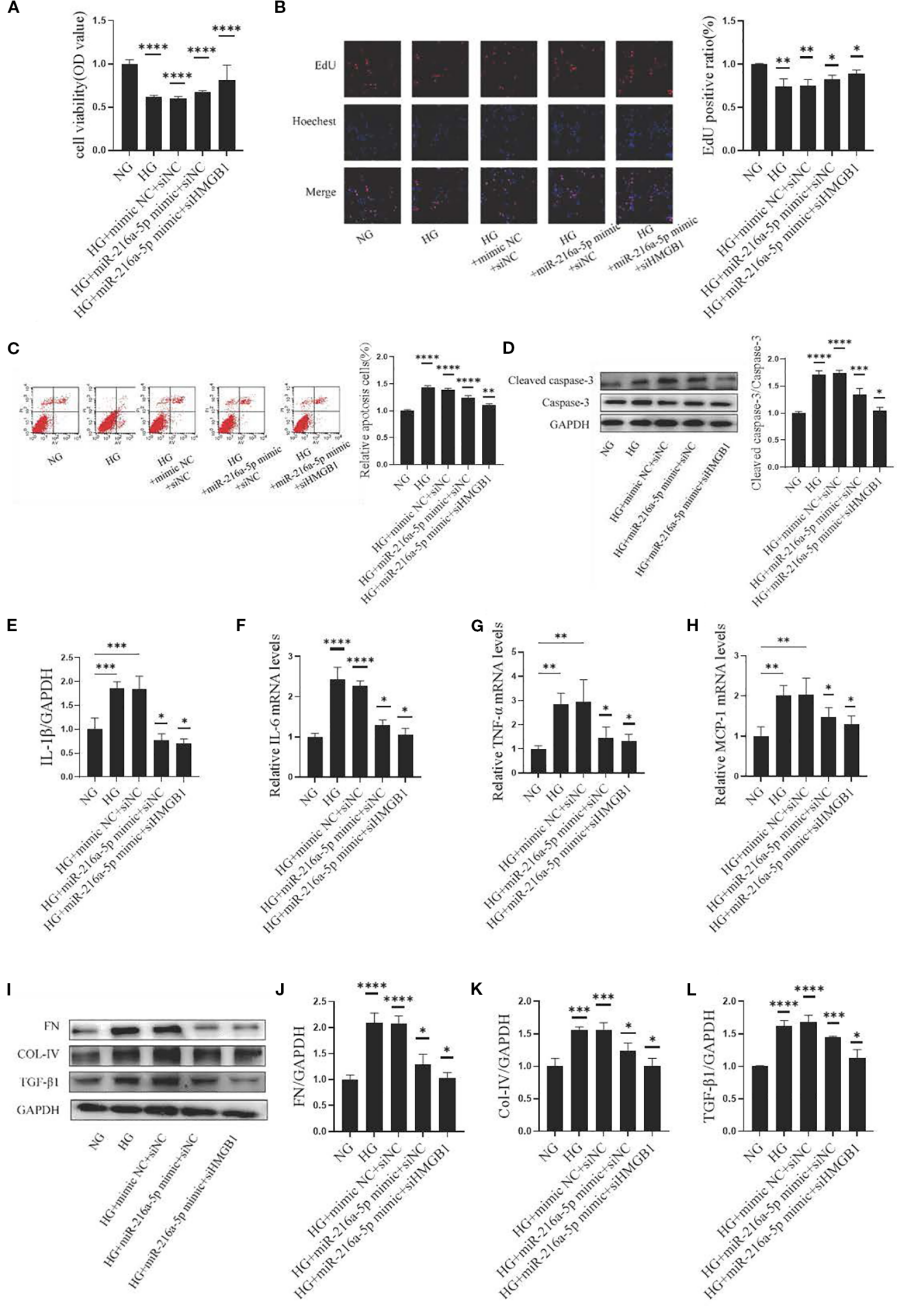
MiR-216a-5p targets HMGB1 to alleviate HMC injury induced by HG. MiR-216a-5p mimics and siHMGB1 were co-transfected into HMC cells. **(A)** Cell viability was detected by the CCK8 method (n=10 per group). **(B)** Cell proliferation was observed by the EdU method (n=3 per group). **(C)** Flow cytometry analysis of apoptosis (n=3 per group). **(D)** Total protein was extracted from the cells and apoptosis-related protein cleaved caspase-3 was analyzed by Western blotting (n=3 per group). **(E-H)** RT-qPCR was used to detect the expressions of inflammatory factors (IL-1β, IL-6, TNF-α and MCP-1) (n=3 per group). **(I-L)** Total cellular proteins were extracted, and fibrotic indicators (FN, COL-IV and TGF-β1) were analyzed by Western blotting (n=3 per group). Data were expressed as mean ± standard deviation. Statistical analysis was performed using univariate ANOVA and Tukey multiple comparison test. *P < 0.05, **P < 0.01, ***P < 0.001, ****P < 0.0001 compared with NG. Abbreviation: NG, Normal glucose (5.5 mmol/L) HG, high glucose (30 mmol/L).

To further explore the relationship between HMGB1 and miR-216a-5p in DN, we co-transfected HMCs with siHMGB1/control and miR-216a-5p mimics/control. The results showed that in the HG+miR-216a-5p mimic+siHMGB1 group, the cell viability increased (*P* < 0.05, **Figure 6A**), the percentage of EdU-positive cells rose (*P* < 0.05, **Figure 6B**), and the proportion of apoptotic cells decreased (*P* < 0.05, **Figures 6C, D**), compared to those in the HG+miR-216a-5p mimic+siNC group. Additionally, co-transfection reduced the expression of inflammatory factors (**Figures 6E–H**) and fibrosis-related proteins (**Figures 6I–L**, *P* < 0.05). These findings indicate that miR-216a-5p may target HMGB1 to inhibit HG-induced cell damage.

### 3.7 MiR-216a-5p offers renoprotection by acting on the HMGB1/RAGE signaling pathway

**Figure 7:**
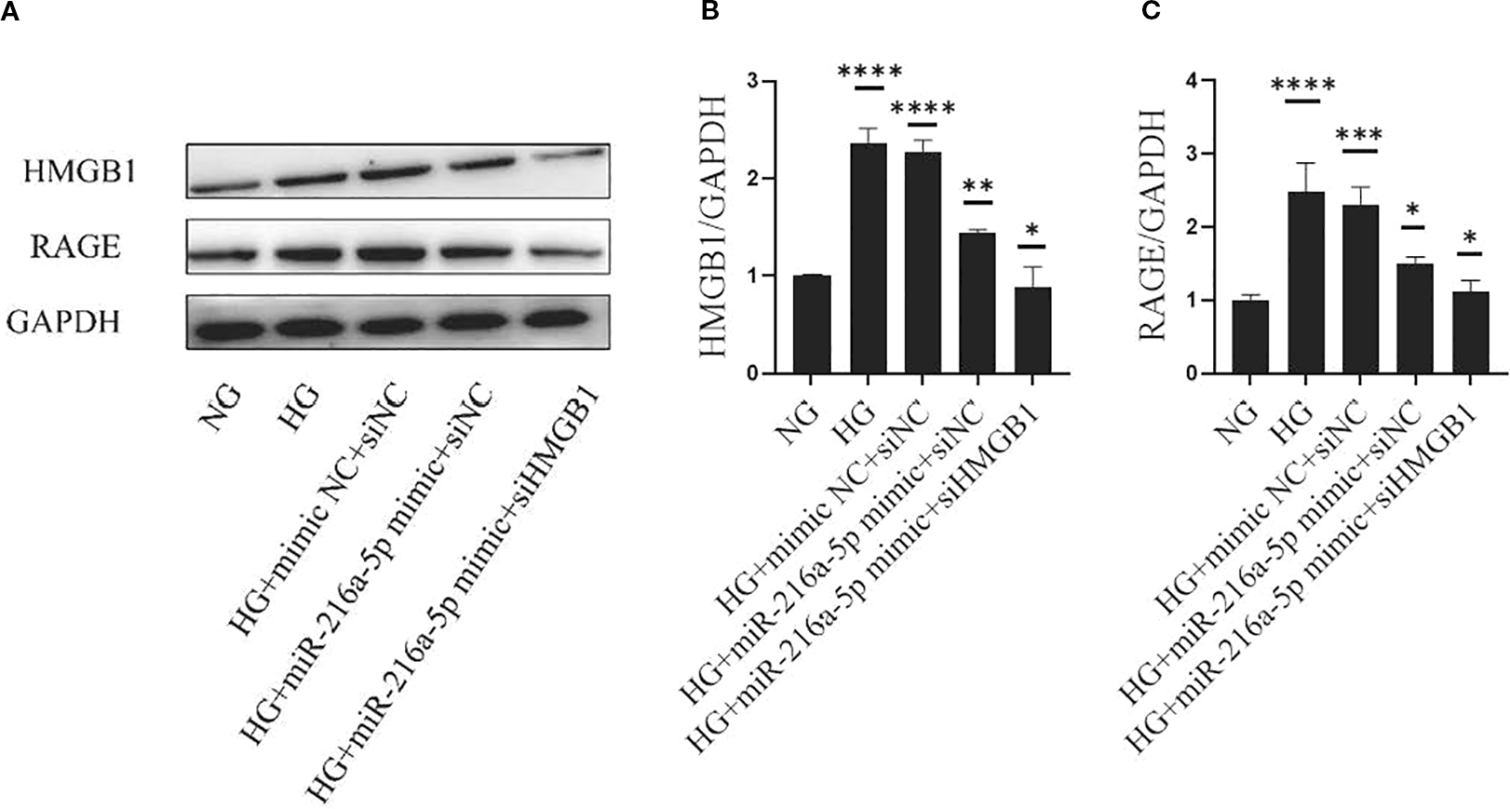
MiR-216a-5p offers renoprotection through the HMGB1/RAGE signaling pathway. Western blotting was used to detect the levels of HMGB1 and RAGE in HMC cells stimulated by HG after co-transfection with miR-216a-5p mimics and siHMGB1. **(A–C)** Total protein was extracted and the expression of HMGB1 and RAGE was analyzed by Western blotting. Data were expressed as mean ± standard deviation. Statistical analysis was performed using univariate ANOVA and Tukey multiple comparison test (n=3 per group). *P < 0.05, **P < 0.01, ***P < 0.001, ****P < 0.0001 compared with NG. NG, Normal glucose (5.5 mmol/L) HG, high glucose (30 mmol/L).

We further clarified the role of RAGE, the receptor of HMGB1, in the renoprotection of miR-216a-5p. As shown in **Figures 7A–C**, after HG stimulation, the protein levels of HMGB1 and RAGE were significantly elevated (*P* < 0.05), and after miR-216a-5p mimic transfection, the expression levels of both HMGB1 and RAGE decreased (*P* < 0.05). After further knockdown of HMGB1, the protein level of HMGB1 decreased, so did its receptor RAGE (*P* < 0.01). The above results suggest that miR-216a-5p may rely on a cascade of HMGB1 and its receptor RAGE to protect renal cells.

## 4 Discussion

In the present study, we verified through cell experiments that miR-216a-5p, a novel miRNA never analyzed in the context renal diseases, could protect against HG-induced HMC injury by targeting the HMGB1/RAGE pathway, thus holding promise as a candidate for further investigation.

Often insidious, DN has already progressed to the macroalbuminuria stage at diagnosis, where the risk of further deterioration into ESRD increased by 14 times, seriously affecting patients’ survival and quality of life (17, 18). Previous studies have found HMGB1 and its receptor RAGE are highly expressed in DN kidneys and co-localize with inflammatory cells, but the mechanisms regulating HMGB1 remain unclear (11). Notably, miR-216a-5p is expressed in HMCs, but downregulated by HG, and this expression was negatively correlated with HMGB1 levels. Mechanistically, we first demonstrated that miR-216a-5p directly targets the HMGB1/RAGE pathway to alleviate inflammation and HG-induced HMC injury, providing a new insight into the pathogenesis of DN.

As non-coding small RNA molecules, miRNAs target mRNAs to regulate the expression of nearly 30% of human genes (19, 20). The abnormal expression of miRNAs can drive the development of various diseases, including tumors, cardiovascular and metabolic diseases (21–23). Research via genetic sequencing and *in vitro* and *in vivo* experiments has established a close link between miRNAs and renal fibrosis in DN (12–16). Furthermore, our study has further provided evidence that miR-216a-5p is closely implicated in DN pathophysiology. Given its role in different stages of DN, miR-216a-5p is expected as a biomarker for the diagnosis and treatment of DN. Meanwhile, miR-216a-5p may be combined with HMGB1 protein and sRAGE (soluble RAGE) to construct a multivariate predictive model to individualize the management for patients with simple DN or complicated with cardiovascular diseases or obesity in clinical scenarios.

HMGB1, a non-histone chromosomal protein in eukaryotic nuclei, is widely distributed in mammals. Extracellular HMGB1 arouses inflammation, drives cell differentiation, and promotes tumor growth (24–26). RAGE, a key HMGB1 receptor, combines with HMGB1 to activate downstream pathways like NF-κB and MAPK, leading to oxidative stress, inflammation, and apoptosis (5–8). Our prior research showed the HMGB1 inhibitor GA can alleviate the damage and inflammation in the kidney of diabetic rats by activating RAGE/TLR4-associated ERK and p38 MAPK/NF-κB (11), suggesting that the HMGB1/RAGE axis is crucial in DN pathology (9, 10). In this study, through dual-luciferase reporter assays, qPCR, Western blotting, we confirmed that miR-216a-5p directly binds to the 3’UTR of HMGB1, consequently inhibiting its expression to curb the development of DN. Our findings prove the complexity of DN pathogenesis and expand the current understanding about this disease.

In summary, our study clarifies the role of the miR-216a-5p/HMGB1/RAGE axis in DN, offering primary experimental evidence for developing miRNA-based precision therapies. However, this study also has relative deficiencies. On the one hand, our research lacks *in vivo* model validation. Subsequently, we plan to create miR-216a-5p knockout mice and validate the mechanism of this regulatory axis in db/db mice and STZ-induced diabetes models. On the other hand, in the existing clinical specimen bank of diabetes, we excluded the influence of other factors, such as heart and kidney diseases, and randomly included patients in three groups: healthy controls, diabetes, and diabetic nephropathy. Due to the initial exploration of changes in miRNA levels *in vivo*, the sample size we included was relatively small, and only variables such as comorbidities and the use of heart and kidney protective drugs were controlled. No special requirements were made for physiological factors such as age and gender. In addition, the currently established sample banks were all collected from inpatients in our hospital, which might have caused certain errors due to selection bias. Finally, due to the sample size, we did not further subgroup the patients in the DN group based on the clinical stage. Therefore, we failed to further verify the role of miR-216a-5p in the different stages of DN course. In the future, we will expand the sample size, and explore the functional transformation of miRNA/HMGB1from pro-inflammatory to pro-fibrotic) as DN progresses, using *in vivo* animal models and human renal tissue biopsy specimens as appropriate.

.

## Data Availability

The raw data supporting the conclusions of this article will be made available by the authors, without undue reservation.
