# Supplementary material for: MiR-216a-5p protects against high glucose-induced HMC injury by targeting the HMGB1/RAGE signaling pathway
Source: Front Endocrinol (Lausanne). 2025 Oct 14;16:1669791. doi: 10.3389/fendo.2025.1669791 (PMC12558729; doi:10.3389/fendo.2025.1669791)
Supplement: Supplementary file 1 [file DataSheet1.docx]

## Materials

The materials involved in this study were presented as follows: DMEM medium (Gibco, USA), fetal bovine serum (Gibco, USA), trypsin (Sigma, USA), HMC cells (Shanghai Cell Bank, Chinese Academy of Sciences). Caspase3 antibody (Abcam, UK), cleaved caspase3 antibody (Abcam, UK), Type IV collagen (collage IV, Col-IV) antibody (Abcam, UK), FN antibody (CST, USA) TGF-β1 antibody (CST Corporation, USA), HMGB1 antibody (Proteintech Corporation, USA), RAGE antibody (Proteintech Corporation, USA) glyceraldehyde-3-phosphate dehydrogenase (GAPDH) antibody (CST Corporation, USA) horseradish peroxidase (HRP)-labeled sheep anti-mouse secondary antibody, HRP-labeled sheep anti-rabbit secondary antibody (Proteintech Corporation, USA), BCA protein concentration determination kit (Shanghai Biyantian Biotechnology Co., LTD.) Youdaoplaceholder0 luminescence (ECL) (Millipore, USA). qPCR primers (Invitrogen Corporation, USA), ChamQ SYBR qPCR Master Mix (Nanjing Novozyme Biotechnology Co., LTD.), miR215a-5p reverse transcription kit (Guangzhou Ruibo Biotechnology Co., LTD.). miR-216a-5p mimics and inhibitors (Guangzhou Ruibo Biotechnology Co., LTD.), HMGB1-siRNA (Suzhou Jima Biotechnology Co., LTD.), Lipofectamine 3000 transfection reagent (Invitrogen Corporation, USA). CCK8 Kit (Nanjing Novozyme Biotechnology Co., LTD.), EdU Kit (Nanjing Jiancheng Bioengineering Graduate Student), Apoptosis Detection Kit (US Everbright Company, USA), Luciferase Reporter Kit (Guangzhou Ruibo Biotechnology Co., LTD.). miRcute Serum/Plasma miRNA Isolation Kit (Beijing Tiangen Biochemical Technology Co., LTD.), miRcute Plus miRNA First-Strand cDNA Kit (Beijing Tiangen Biochemical Technology Co., LTD.), miRcute Plus miRNA qPCR Kit (SYBR Green) (Beijing Tiangen Biochemical Technology Co., LTD.). Enzyme-linked Immunosorbent Assay Kit (HMGB1, IL-1β, IL-6, TNF-α, MCP-1) (Shanghai Jianglai Biotechnology Co., LTD.). The HMC cells were purchased from the American Type Culture Collection (ATCC, Manassas, VA, USA).

## Clinical serum samples

Data were collected following related protocols, including double data entry and verification, and regular quality control. According to the inclusion and exclusion criteria, a specimen bank for diabetic nephropathy was established after eligible patients were enrolled (Table 1). The remaining serum of healthy patients was collected from the physical examination center to form a specimen bank of healthy people.

Table 1 The inclusion and exclusion criteria.

| Specimen bank for patients with diabetic nephropathy |
| --- |
| Inclusion criteria:   - Aged between 18 and 70. - Meeting the World Health Organization's diagnostic criteria for type 2 diabetes in 1999. - Diagnostic criteria for diabetic nephropathy: According to the "Chinese Guidelines for the Prevention and Treatment of Diabetic Nephropathy (2021 Edition)", the urinary albumin/creatinine ratio (ACR) of diabetic patients is higher than 30mg/g and/or the glomerular filtration rate is lower than 60 ml·min^-1^· (1.73m^2^) ^-1^ for more than 3 months, while excluding chronic kidney disease caused by other reasons. |
| Exclusion criteria:   - Type 1 diabetes, gestational diabetes or other special types of diabetes; - Presence of other kidney diseases such as purpura nephropathy, mesangial proliferative glomerulonephritis, IgA nephropathy or nephrotic syndrome, etc. - Presence of severe cardiovascular diseases such as old myocardial infarction, acute heart failure, acute cerebral infarction, acute cerebral hemorrhage, and lower extremity arterial occlusion, etc. - Presence of malignant tumors, hematological diseases and severe digestive system diseases, intractable hypertension, gout, severe hyperlipidemia, etc. - Histories of hypoglycemic coma, hyperosmolar coma, severe infection, surgery, trauma or other stress states in the past three months. - Previous uses of glucocorticoids, diuretics, and aldosterone antagonists in the past three months. |

Accordingly, patients with neurodegenerative diseases such as Alzheimer's disease were further excluded. In the conventional hypoglycemic regimen, cardiovascular protective drugs including SCLT2 inhibitors and kidney-protecting drugs including RAS system inhibitors were excluded. Ultimately, three groups were constructed for this study (Table 2): healthy control group (Control, n=9), the diabetic group (DM, n=16), and the diabetic nephropathy group (DN, n=21).

Table 2 The demographic, clinical, and laboratory profiles of all the subjects.

| Variables | healthy control group (n=9) | DM group (n=16) | DN group (n=21) |
| --- | --- | --- | --- |
| Age (years) | 50.0 (44.5-58) | 56.5 (53.0-68.5) | 58.0(48.5-67.5) |
| Sex, male/total (%) | 33.3 | 81.3 | 42.9 |
| Duration of diabetes (years) | - | 4.5 (1.3-10.8) | 18 (8-21) |
| BMI (kg/m^2^) | 22.7 (3.3) | 24.1 (2.7) | 27 (3.9) |
| Systolic blood pressure (mmHg) | 121 (8) | 130 (11) | 137 (9) |
| Diastolic blood pressure (mmHg) | 75(7) | 77 (9) | 80 (7) |
| HbA_1c_ (%) | 5.5 (0.3) | 7.5 (1.3) | 9.7 (2.3) |
| Fasting glucose | 4.99 (0.32) | 6.43 (1.71) | 9.19 (4.21) |
| Plasma total cholesterol (mmol/L) | 4.23 (0.96) | 3.85 (1.08) | 4.74 (1.30) |
| Plasma HDL cholesterol (mmol/L) | 1.49 (0.51) | 1.12 (0.24) | 0.98 (0.24) |
| Plasma LDL cholesterol (mmol/L) | 2.60 (0.67) | 2.25 (0.99) | 3.01 (1.18) |
| Plasma triglycerides (mmol/L) | 1.20 (0.53) | 1.30 (0.72) | 2.13 (1.68) |
| Blood urea nitrogen (mmol/L) | 4.47 (0.77) | 5.76 (1.18) | 8.53 (4.34) |
| Serum creatinine (mmol/L) | 62.10 (54.75-65.90) | 71.90 (60.65-84.05) | 73.90 (69.30-104.85) |
| Serum uric acid (mmol/L) | 256 (216-344) | 313 (264-364) | 345 (282-415) |
| Urinary ACR (mg/g) | 0.64 (0.27-1.30) | 3.01 (2.14-3.34) | 287.12 (73.06-914.32) |
| eGFR (ml·min^-1^·（1.73m^2^）^-1^） | 104.2 (4.7) | 92.2 (12.3) | 76.7 (29.0) |

All values are mean (SD) for normally distributed data and median (interquartile range 25-75%) for skewed data. HbA_1c_ is reported as percent. Sex data is percentage (%).
