# Supplementary material for: MiR-216a-5p protects against high glucose-induced HMC injury by targeting the HMGB1/RAGE signaling pathway
Source: Front Endocrinol (Lausanne). 2025 Oct 14;16:1669791. doi: 10.3389/fendo.2025.1669791 (PMC12558729; doi:10.3389/fendo.2025.1669791)

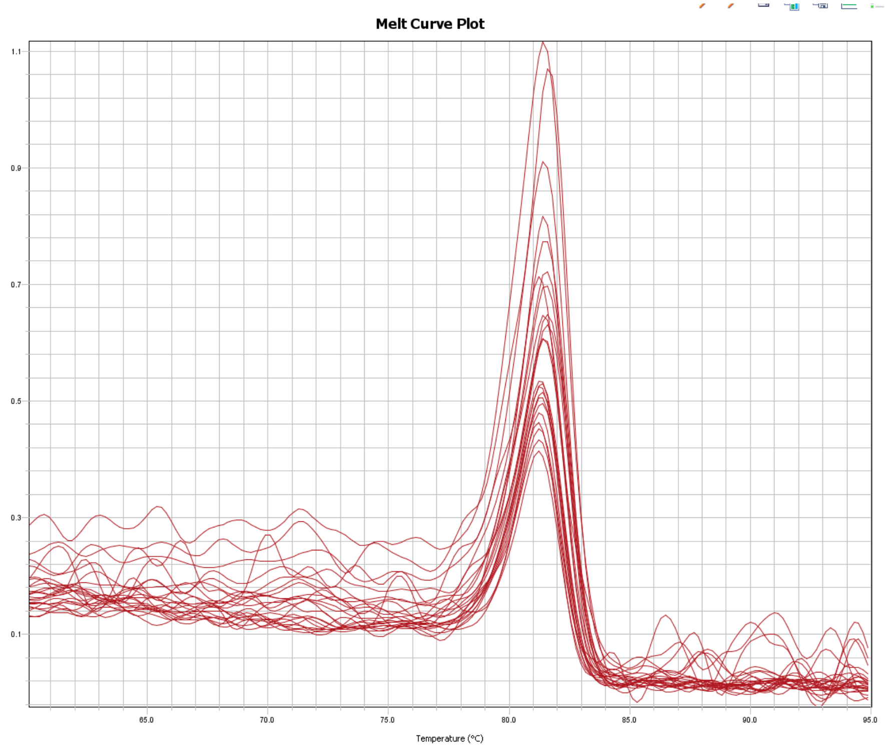

miR-216a-5p溶解曲线

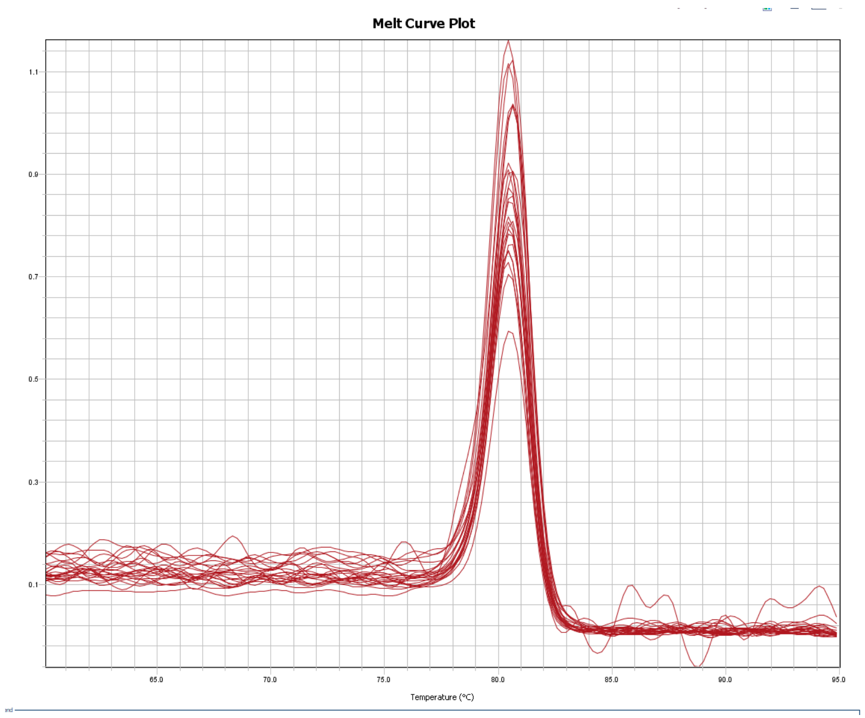

U6溶解曲线

数值见Excel表

细胞活力

	1	2	3	4	5	6	7	8	9	10	11	12	
A	0.048	0.048	0.048	0.048	0.048	0.049	0.048	0.048	0.048	0.048	0.048	0.048	450
B	0.048	2.018	2.329	2.45	2.472	2.48	2.427	2.439	2.357	2.32	1.994	0.048	450
C	0.048	1.35	1.254	1.314	1.277	1.281	1.336	1.301	1.352	1.249	1.351	0.048	450
D	0.048	1.286	1.297	1.267	1.415	1.304	1.324	1.279	1.285	1.333	1.203	0.048	450
E	0.048	1.38	2.011	2.082	2.039	2.086	2.073	2.037	1.953	1.942	0.894	0.047	450
F	0.049	1.268	1.191	1.326	1.33	1.195	1.22	1.268	1.378	1.274	1.272	0.047	450
G	0.048	1.281	1.252	1.18	1.226	1.185	1.209	1.197	1.145	1.077	1.212	0.048	450
H	0.048	0.049	0.049	0.048	0.048	0.048	0.048	0.048	0.048	0.049	0.048	0.048	450

	1	2	3	4	5	6	7	8	9	10	11	12	
A	0.048	0.048	0.051	0.048	0.048	0.048	0.048	0.048	0.048	0.048	0.048	0.048	450
B	0.051	2.391	2.569	2.688	2.644	2.679	2.566	2.588	2.63	2.558	2.323	0.048	450
C	0.048	1.554	1.611	1.492	1.578	1.654	1.607	1.603	1.626	1.475	1.552	0.048	450
D	0.048	1.576	1.535	1.625	1.611	1.521	1.479	1.458	1.424	1.556	1.512	0.048	450
E	0.047	1.716	1.757	1.689	1.688	1.775	1.758	1.717	1.748	1.738	1.749	0.047	450
F	0.048	1.536	2.289	2.282	2.367	2.334	2.253	2.267	2.284	2.152	0.982	0.048	450
G	0.049	0.048	0.048	0.048	0.048	0.048	0.047	0.047	0.048	0.048	0.048	0.048	450
H	0.048	0.048	0.048	0.048	0.047	0.048	0.047	0.047	0.048	0.048	0.048	0.048	450

荧光素酶

原始荧光值	Luc	h-HMGB1-WT+NC	h-HMGB1-WT+hsa-miR-216a-5p	h-HMGB1-MUT+NC	h-HMGB1-MUT+hsa-miR-216a-5p
	1	1212566	1069731	2080791	1660467
	2	972096	1179574	1871434	1722331
	3	1019469	954751	2058765	1954105
	Rluc	h-HMGB1-WT+NC	h-HMGB1-WT+hsa-miR-216a-5p	h-HMGB1-MUT+NC	h-HMGB1-MUT+hsa-miR-216a-5p
	1	697323	286558	1223575	921828
	2	497135	306130	1102940	970037
	3	493855	233515	1177771	1156903
荧光校正	Rluc/Luc	h-HMGB1-WT+NC	h-HMGB1-WT+hsa-miR-216a-5p	h-HMGB1-MUT+NC	h-HMGB1-MUT+hsa-miR-216a-5p
	1	0.58	0.27	0.59	0.56
	2	0.51	0.26	0.59	0.56
	3	0.48	0.24	0.57	0.59
	Ave	0.52		0.58	
相对荧光值		h-HMGB1-WT+NC	h-HMGB1-WT+hsa-miR-216a-5p	h-HMGB1-MUT+NC	h-HMGB1-MUT+hsa-miR-216a-5p
	1	1.10	0.51	1.01	0.95
	2	0.98	0.50	1.01	0.97
	3	0.93	0.47	0.98	1.02
	Ave	1.00	0.49	1.00	0.98

分组	管号	姓名	肌酐 (umol/L)	miR-216a-5p	IL-6	IL-1β	TNF-α	HMGB1	MCP-1
健康对照组 (9)	60	欧建红	65.8	1.022	1.421626	8.184463	10.37107	228.6512	485.2872
	4006	吴秀梅	62.1	1.014	1.445839	8.587059	8.784253	234.9695	553.9098
	37	顾殿华	73	1.026	1.854777	7.564554	8.20567	228.951	227.157
	249	支秀兰	49	1.002	0.993667	8.761526	11.88087	289.9744	652.4556
	235	李凤梅	60.5	0.998	2.784759	9.711468	12.65529	307.671	671.3663
	4009	张中成	66	0.984	2.599756	10.13735	9.044303	268.8918	508.4419
	138	吴兰芳	63.4	0.988	1.908653	10.01503	11.05085	238.6016	553.9098
	48	严红	46.6	1.062	1.828064	8.471389	9.702186	198.8851	596.0934
	4037	赵开宏	61.1	0.94	2.974388	10.44537	12.4428	306.0147	673.8922
DM组 (16)	90	杨刚	81.5	0.698	2.479054	14.52859	10.7096	278.722	762.9034
	147	钱万祥	89.6	0.682	4.059056	16.67946	17.72543	303.0427	794.9569
	63	程广平	58.7	0.664	6.014425	21.48837	17.88182	410.3408	1079.091
	49	于本信	91.6	0.724	2.018175	11.32474	8.979124	238.6016	648.6805
	51	杨永亮	73.7	0.786	1.594499	10.69407	8.525986	217.6314	639.8816
	60	张成驻	83.9	0.722	2.07381	11.70924	8.914056	262.6154	689.0684
	19	李前岭D	60	0.514	6.636389	33.48908	35.75974	530.5857	1169.072
	30	顾太祥	87.3	0.682	2.630264	18.86755	13.01156	329.5449	805.2409
	52	吴世民	59.5	0.66	5.933014	21.81556	18.8288	415.6992	1098.893
	89	张伯志	74.7	0.674	4.384958	16.75313	17.96017	339.5168	874.9768
	80	李长领	62.6	0.686	3.267237	14.80877	16.33646	271.7328	847.7934
	79	王翠霞	58.2	0.663	5.851967	23.22919	18.43243	378.258	1114.754
	36	肖正理	63.8	0.642	6.594311	29.07441	24.73156	496.403	1116.077
	23	张莉	66.9	0.702	2.389937	11.9032	9.437707	234.3657	695.4024
	55	王金星	70.1	0.74	1.8015	11.3885	8.719519	230.1512	643.6509
	103	潘志鹍	84.1	0.694	2.244144	14.45886	12.16098	283.8453	762.9034
DN组（21）	8	吉玉凤	154.7	0.382	8.387181	35.19258	20.68498	300.0825	1077.771
	11	吴菊博	71.8	0.394	6.848062	29.8219	18.66994	279.0412	980.5147
	21	陈彤耀	68.4	0.402	5.139387	27.96909	14.68445	278.4029	954.3605
	41	欧阳红	68.8	0.428	5.531478	25.99196	13.44259	265.1199	829.7156
	44	何锦春	113.4	0.384	8.071596	33.39009	20.52143	332.6252	1089.648
	70	王昌洪	85	0.366	10.55003	57.23174	38.15485	434.3736	1240.887
	43	左留兆	73.9	0.228	18.04839	69.70552	106.8139	634.6332	2103.304
	93	高苏华	73.7	0.414	5.062133	26.97257	14.24267	272.9989	946.5259
	124	谢守华	86.1	0.364	10.99023	55.69887	36.85039	497.2604	1118.722
	123	李美元	63.4	0.372	8.844442	44.41822	26.75276	369.5502	1105.5
	138	倪玮	54.8	0.353	11.78568	67.30292	41.54166	563.1037	1600.959
	148	赵兴玉	64.5	0.334	14.42432	66.04909	69.83652	597.5541	1667.313
	124	谢守华	74.9	0.368	9.971495	40.75687	30.76692	474.4516	1108.143
	1	范金玲	142.1	0.38	8.614878	30.10439	24.73156	313.9984	1031.68
	146	单花猛	73.9	0.346	13.73748	59.30891	59.26309	547.3582	1405.471
	32	刘立芳	206.7	0.402	6.594311	28.06047	15.65454	279.68	938.6969
	152	顾祝琴	74.9	0.392	7.848498	31.53488	18.43243	283.203	979.2055
	26	鲁正梅	69.8	0.398	7.147952	28.33541	18.11718	280.9592	979.2055
	119	葛恒芳	124.2	0.386	8.071596	37.97897	24.04099	299.4262	1027.737
	54	蒋其明	69.8	0.37	10.21137	40.86559	30.30103	305.3532	1203.612
	114	邢正兴	96.3	0.418	6.260823	27.24277	14.38951	276.8095	838.75

原图3重复

目的蛋白
HMGB1

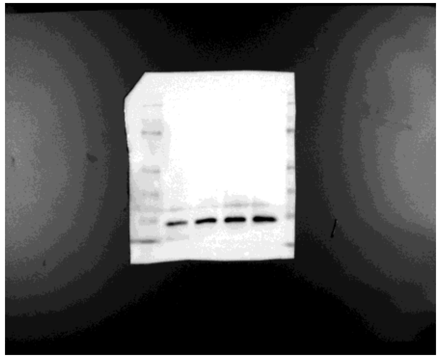

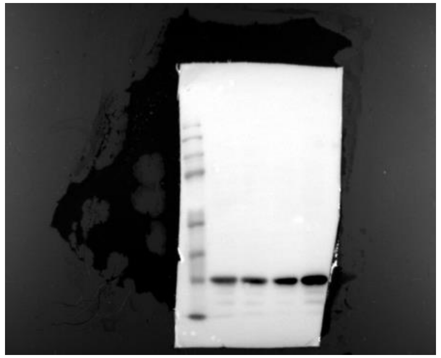

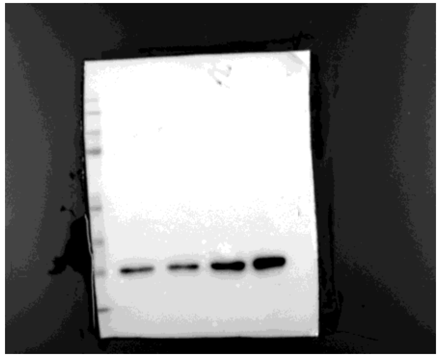

内参
GAPDH

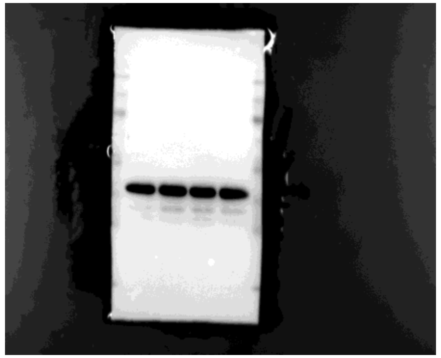

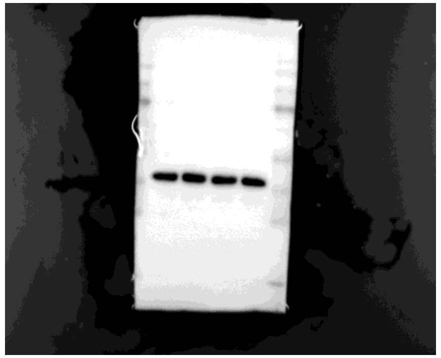

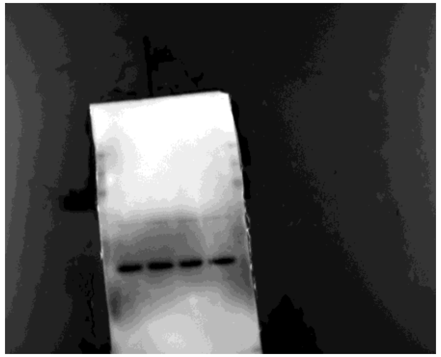

原图3重复

目的蛋白
cleaved caspase3

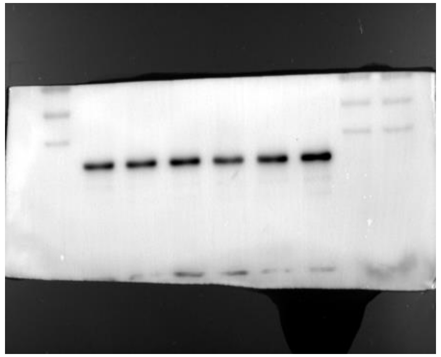

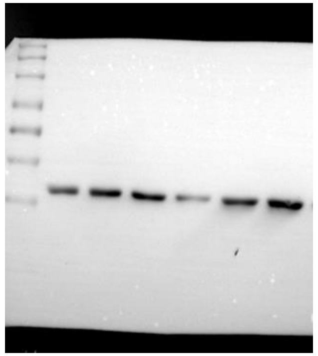

目的蛋白
caspase3

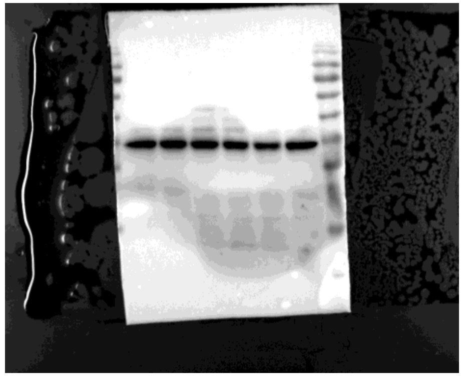

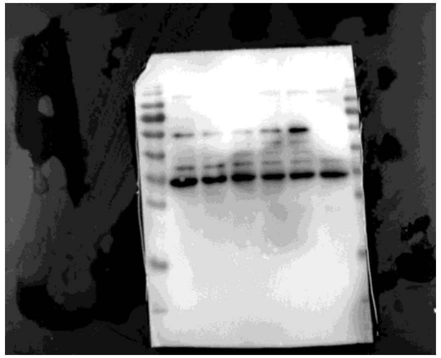

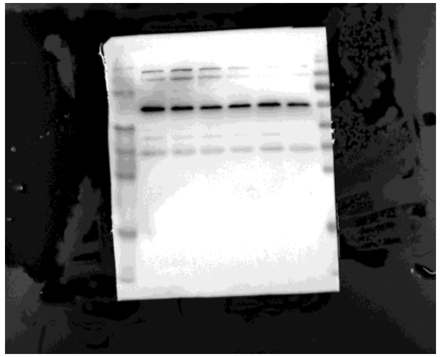

内参
GAPDH

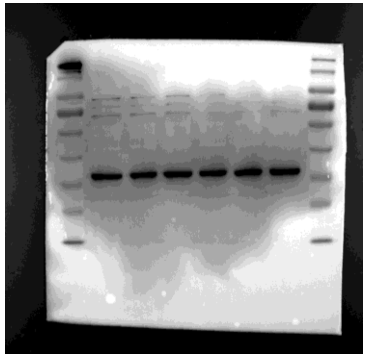

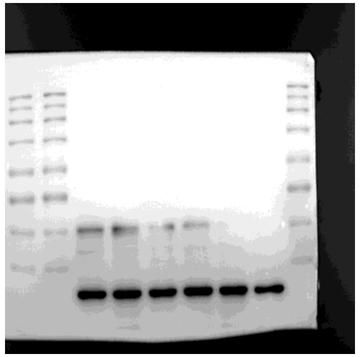

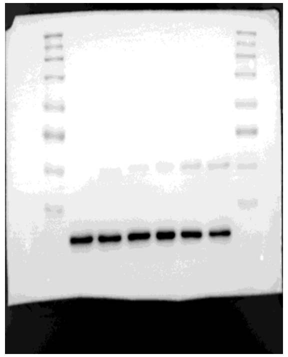

原图3重复

目的
蛋白
FN

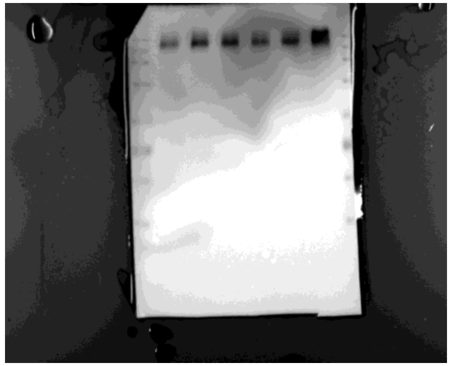

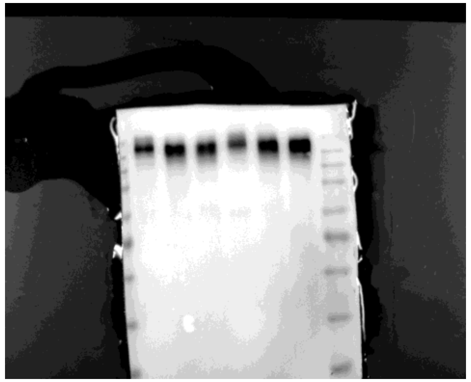

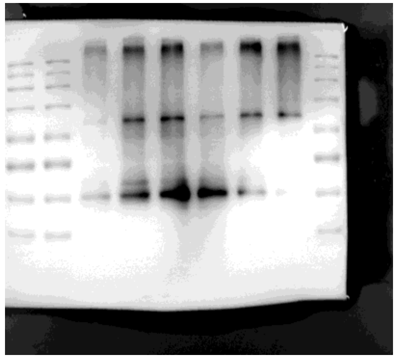

目的
蛋白
col iv

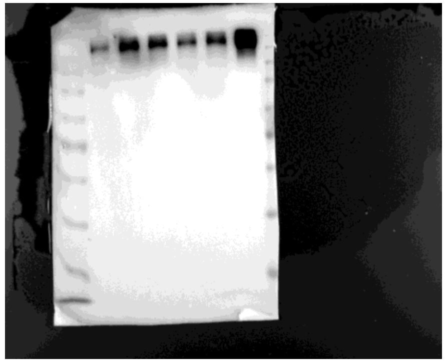

目的
蛋白
tgf

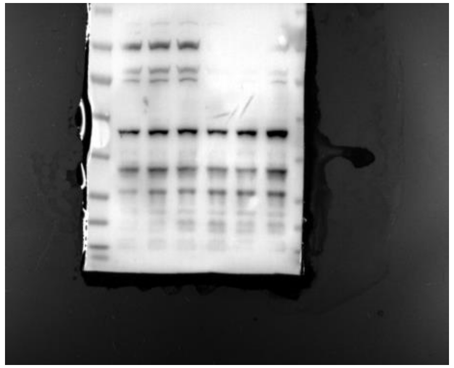

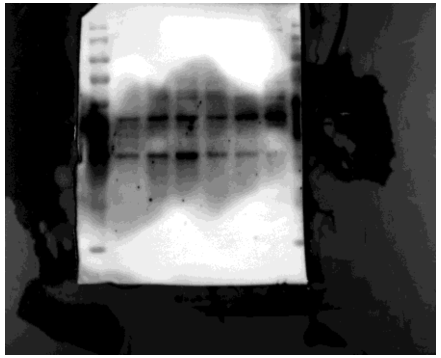

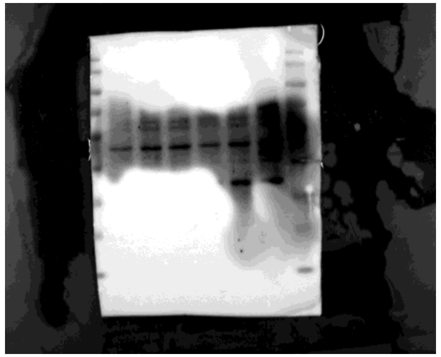

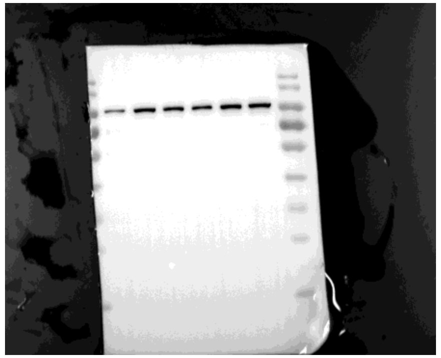

内参
GAPDH

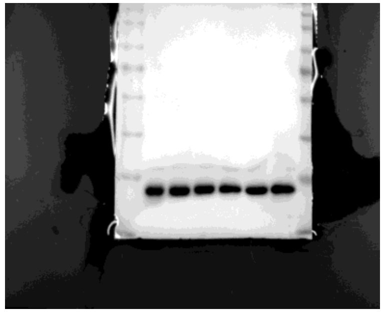

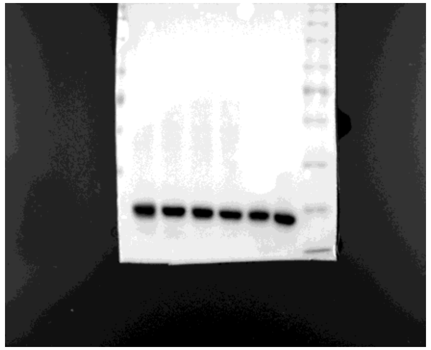

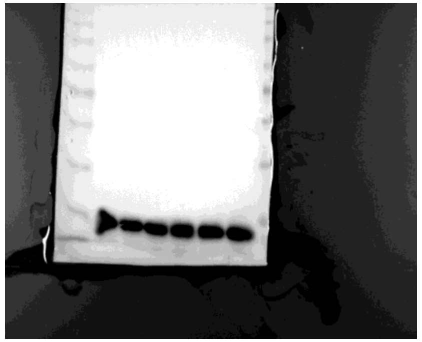

原图3重复

目的蛋白
HMGB1

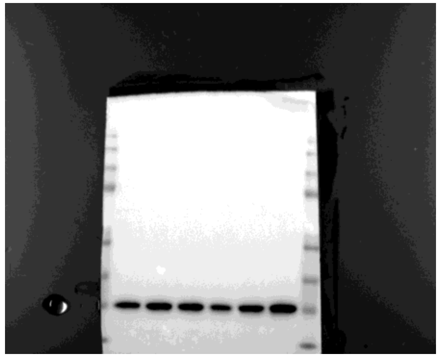

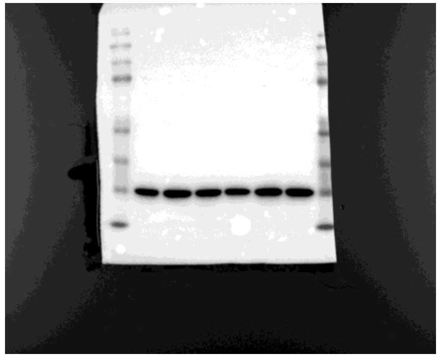

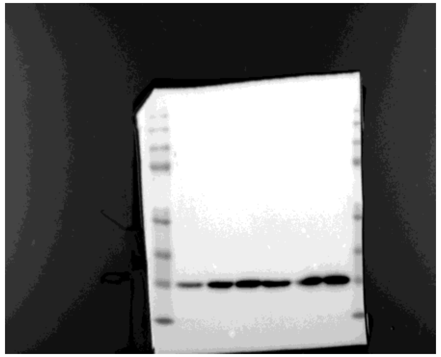

内参
GAPDH

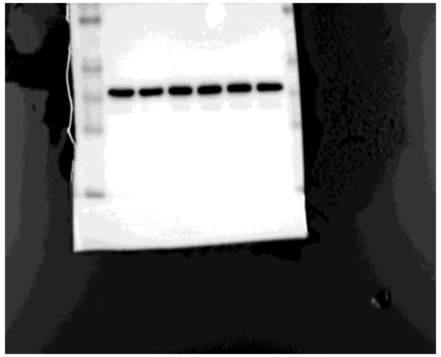

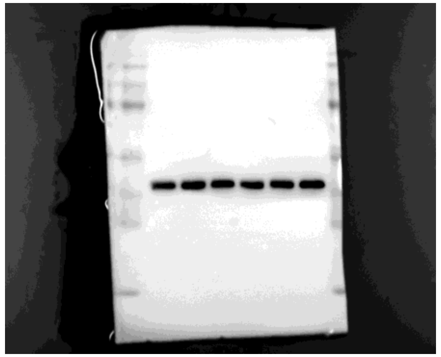

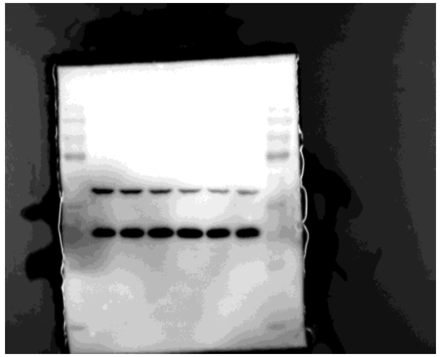

原图3重复

目的蛋白
cleaved
caspase3

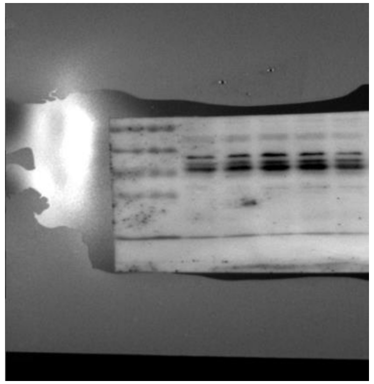

目的蛋白
caspase3

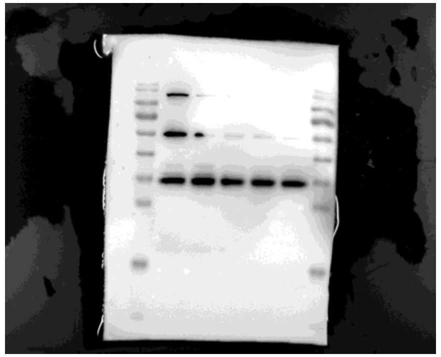

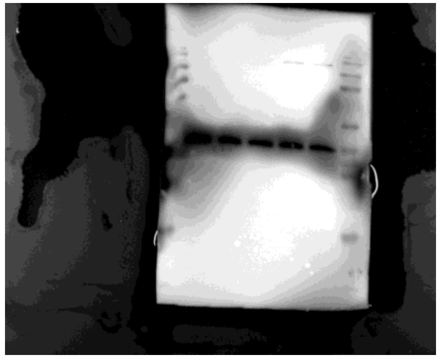

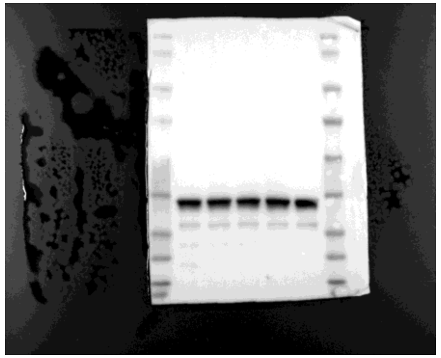

内参
GAPDH

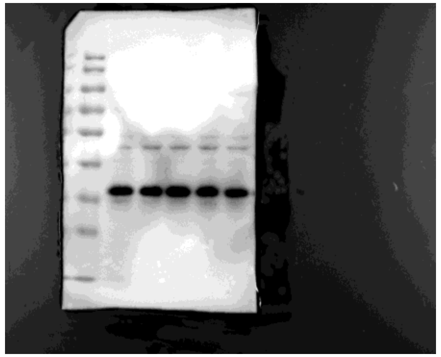

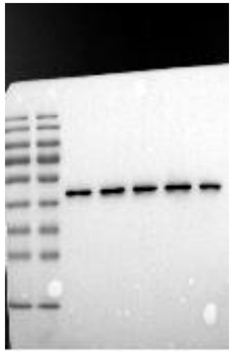

原图3重复

目的
蛋白
col iv

目的
蛋白
FN

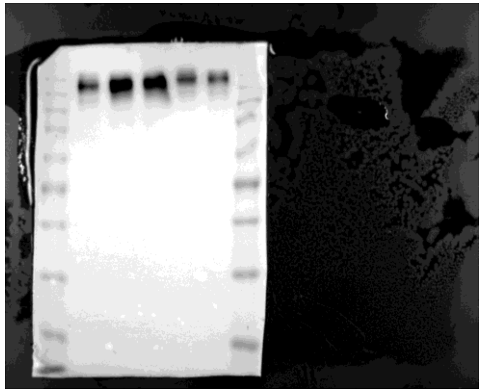
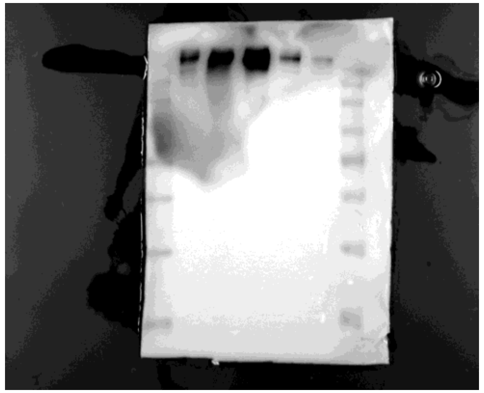
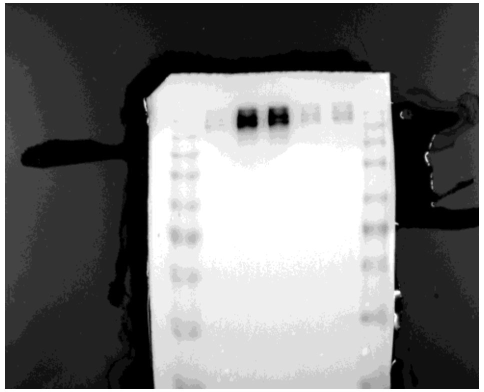
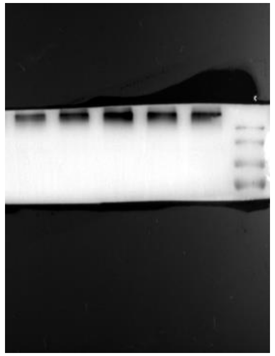

目的
蛋白
tgf

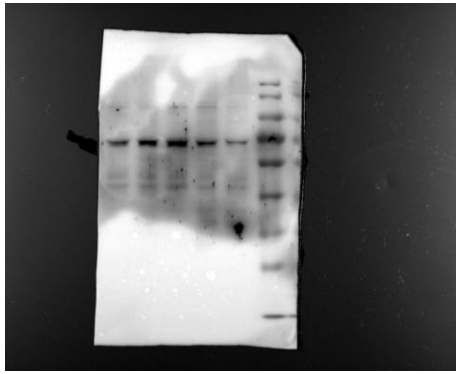
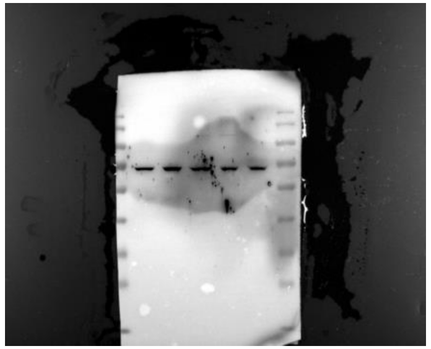
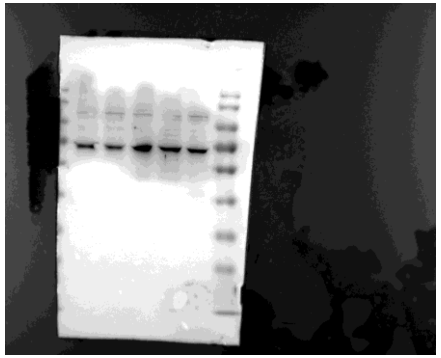
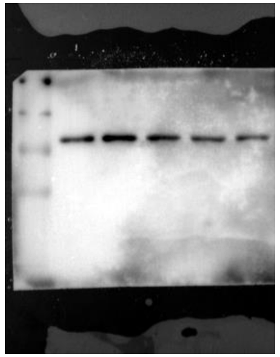

内参
GAPDH

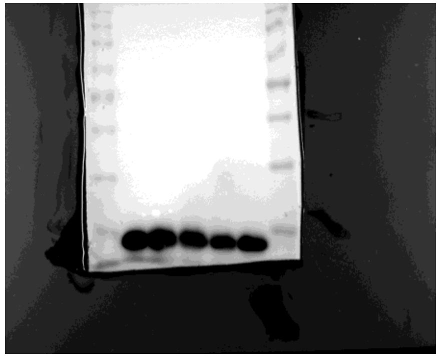
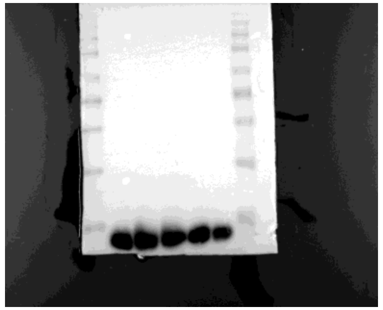
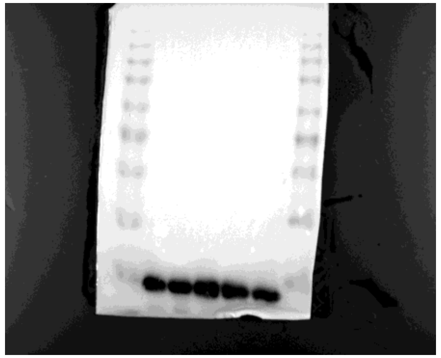

原图3重复

目的蛋白
HMGB1

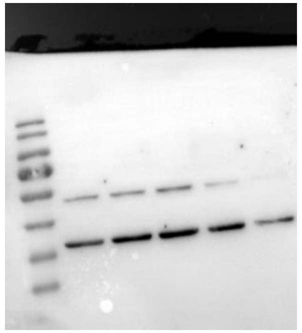

目的
蛋白
RAGE

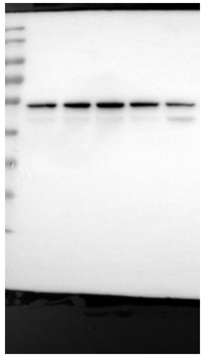

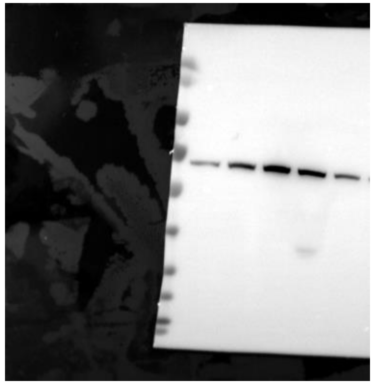

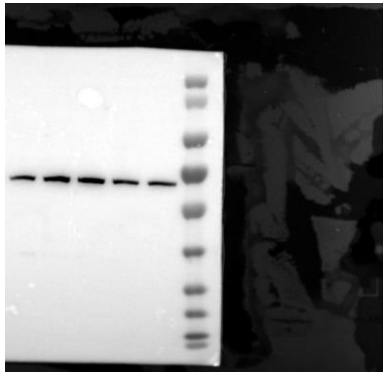

内参
GAPDH

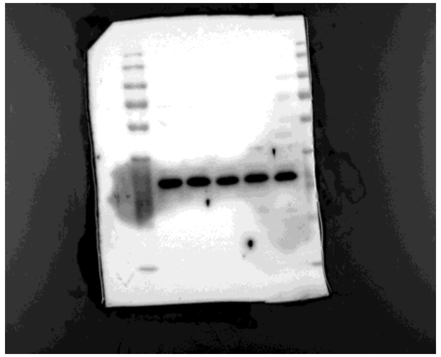

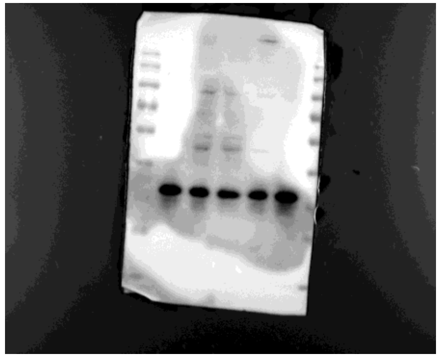

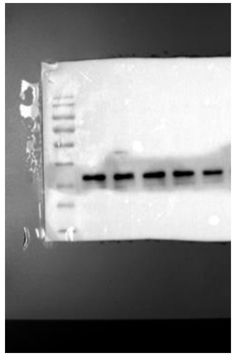

Supplement: Supplementary file 2 [file DataSheet2.pdf]
